# A Way to Understand Inpatients Based on the Electronic Medical Records in the Big Data Environment

**DOI:** 10.1155/2017/9185686

**Published:** 2017-02-09

**Authors:** Hongyi Mao, Yang Sun

**Affiliations:** ^1^Economics and Management School, Jiujiang University, Jiujiang 332005, China; ^2^Union Hospital, Tongji Medical School, Huazhong University of Science and Technology, Wuhan 430022, China

## Abstract

In recent decades, information technology in healthcare, such as Electronic Medical Record (EMR) system, is potential to improve service quality and cost efficiency of the hospital. The continuous use of EMR systems has generated a great amount of data. However, hospitals tend to use these data to report their operational efficiency rather than to understand their patients. Base on a dataset of inpatients' medical records from a Chinese general public hospital, this study applies a configuration analysis from a managerial perspective and explains inpatients management in a different way. Four inpatient configurations (valued patients, managed patients, normal patients, and potential patients) are identified by the measure of the length of stay and the total hospital cost. The implications of the finding are discussed.

## 1. Introduction

In recent decades, the influence of information technology (IT) in healthcare has drawn great attention from both practical and academic worlds [1, 2]. Billions of money have been spent on healthcare IT projects to increase care coordination, improve service quality, and reduce costs in hospitals [3]. However, the healthcare industry is still challenged by the rising cost and the government regulation decisions [4], which leads to the contradiction between the patients and the healthcare providers. The conflict has become a serious social problem in China. Though healthcare IT seems to be a very important approach to solve this problem, the use of IT in Chinese hospitals remains on the initial stage, and the value of generated data has not been fully discovered.

Electronic Medical Record (EMR) or Electronic Health Record system stores the medical history of patients [3], including the admission note, the progress notes, the treatment orders, the surgery information, the lab and other examination results, and the summary of medical record including the costs information. Adoption of EMR system could improve healthcare service and documentation, acting as a knowledge base for hospitals [5]. Millions of medical records have been generated with the continuous use of EMR, drawing growing interest for scholars in both healthcare and information system disciplines to conduct relevant studies with the big dataset. For scholars in healthcare, EMR system is a valuable tool to identify and optimize treatments by enabling the efficient retrieval of the medical history of a patient for a certain disease [6]. The information stored in the system is also vital for hospital managers to evaluate the operation and to guide the decisions. For information system researchers, EMR is an IT project for evaluating and predicting certain variables, such as the risks, the hospital performance, and the service level [7]. In both ways, EMR system supports effective and efficient hospital operations [8].

Synchronized with the worldwide adoption and application of the EMR systems, the Chinese government has published a relevant standard requiring every public hospital to adopt an EMR system to create, store, organize, and report inpatients' medical history to the National Statistical Offices [9]. This policy has made it easier to aggregate inpatients' data [10]. However, the dataset has not been fully used by Chinese hospitals. The records from EMR system in Chinese hospitals are used to monitor the quality of the hospital [11] and to calculate the Diagnosis Related Groups (DRG) of the patients for ranking and insurance reimbursement purposes [12]. The published rankings could help the hospitals to attract more patients. However, the efficient service and management of increasing amount of patients are neglected and leave a huge space for further discussion. It seems that Chinese hospitals use these datasets to report rather than to guide their management. Therefore, a deep analysis of records in the EMR system for management is required.

An investigation of the EMR is to analyze the patients. Treating patients is the main value-added business of hospitals, making the patients like customers to the hospital. The studies on customer experience, such as the customer segmentation, provide a lot of useful information about how strategies transform into performance [13]. Similarly, the study on patients' segmentation could offer managerial guidelines to improve performance in a hospital. Literature often classifies patients based on the type of diseases [11]. Few studies focus on the managerial aspect. The delivery of the healthcare service for a hospital is more complicated than for other industries. The complexity of human diseases and the individual differences bring high risks and difficulties for hospitals to satisfy their customers [14]. Furthermore, it is sometimes difficult for the managers of hospitals to know how to manage all kinds of patients with different diseases. Therefore, a brief understanding of patients in the managerial perspective is urgent and necessary and could be a way to improve the performance of a hospital.

EMR contains a lot of critical factors for the hospital management. The length of stay and the hospital cost are among the most important factors [15, 16]. These factors are usually used as indicators for hospitals to monitor their performance [15, 17]. For example, scholars devote efforts to predict the length of stay of patients facing certain diseases with different treatment methods [6, 16]. Therefore, with these factors, we could develop a set of stable and relevant configurations of patients in a holistic view. The configuration analysis of the patients could offer a fruitful lens in explaining patients' experience and show a direct way to manage patients. Motivated by the literature gap discussed above, this study aims to examine the following questions in the context of inpatients in a Chinese hospital:

1. How can we define and classify an inpatient configuration?
2. How does the concept of inpatient configurations help to explain and guide management in a different way?

## 2. Research Background

EMR system stores information including the clinical operations, the medical images, the patient behaviors, and the activities from healthcare providers in a variety of formats. The application of EMR system increases the complexity of analyzing healthcare data from perspectives of variety, velocity, and volume [18]. Therefore, EMR becomes a valuable source for scholars to conduct big data analysis for different purposes [19, 20]. Lau et al. [21] find that the use of EMR in healthcare covers topics in healthcare communication, decision-making support, patient outcomes, and hospital performance. The initial use of EMR is to increase the information flow between patients and clinic units to ensure the continuity of healthcare [20]. Then, EMR seems to have transformed healthcare by saving cost for both outpatients and inpatients [8]. Reid [22] suggests to leverage EMR to pare expenses down for healthcare providers because the information stored in the system could significantly reduce redundancy like repeated examinations and provide better care for patients with chronic diseases. Buntin et al. [23] also point out that the implementation of EMR system could help hospitals to mitigate the increasing cost of healthcare. For healthcare providers, EMR is more than a kind of useful IT artifact, increasing the ability to update and retrieve medical data [20]. Linked with social media and cloud computing, EMR becomes a knowledge platform to understand patients with certain diseases and to improve clinical practice [21, 24]. For example, in China, EMR is used to generate and improve the Hospital Quality Monitoring System reports and to optimize the classification of diseases [11]. The continuous renewed clinical data groups could also help the popularization of the EMR system [9]. Though hospitals have used EMR systems to answer healthcare problems for years, patients are rarely involved in the adoption and the related activities of the system, which may impair the value of the system [21].

Scholars have investigated the role of patients to the hospital. The satisfaction and loyalty of patients can significantly influence the performance of healthcare providers [14, 25, 26]. Vogus and McClelland [14] discuss the changes from normal customers to patients and highlight that patient satisfaction and service quality are often difficult to achieve due to the risk and complexity. Turner et al. [27] also point out that it is difficult to achieve a continuous patient-hospital relationship. Therefore, it is necessary to apply customer relationship management in the area of patient management to enhance patient loyalty, eventually leading to high hospital performance [25]. Moreover, patient flow, the movement of patients in healthcare processes, is another important indicator for efficient and effective hospital management [28, 29]. Hall [29] argues that a well-organized patient flow can reduce the delay in the delivery of healthcare service. Patients move through both clinical and operational processes could be very complex; thus a carefully planned and structured patient flow can reduce the congestion and increase the overall performance of the hospital [25]. Therefore, the role of patients is important in management practice in the hospital. It is noteworthy that the standardization and implementation of EMR systems are an approach to rearranging patients' movement.

## 3. Methods and Results

### 3.1. Configuration Analysis

A configuration refers to “a constellation of conceptually distinct elements or traits that commonly occur together and form an integrative meaningful whole” [30, p. 498]. Configuration analysis is to use some distinct but interdependent elements to form a stable and meaningful set of coherent patterns. Therefore, we define inpatient configuration as a set of meaningful and holistic groups of inpatients. The configuration analysis offers a new understanding of the inpatients of the hospitals, especially for those who possess the bulk of high level medical resources. The relationship between the hospital and the different types of patients could be developed in each configuration due to the different combinations of key elements. In order to generate available and meaningful configurations within the given medical records, this study selects the total hospital cost and the length of stay as the key elements. We adopt the hospital cost and the length of stay in the configuration analysis because they are two distinct but interdependent elements that could reflect behaviors of both patients and hospitals.

The hospital cost is a variable that is valued by patients, hospitals, and the society [15]. In this study, this factor refers to the total amount of money spent on healthcare service during one's stay in the hospital. Due to the lack of funding to support the public hospitals, in 2006, the Chinese government proposed an approach allowing all the public hospitals to sell drugs at the 15% higher price to generate revenues. The policy leads to continuous increasing of the hospital costs. Under the circumstances of insufficient healthcare insurance fund, the burden for the patients became tremendous. In 2012, the government decided to gradually terminate the old policy. Facing the change, public hospitals must discover new revenue growth to sustain the development. The associations among hospital cost, healthcare quality, and patient satisfaction have also been discussed in the literature [27, 31]. In this way, the hospital cost becomes an important indicator for hospital operations responding to the changing environment.

The length of stay is another significant and commonly employed indicator for the ability of a hospital [6, 17]. Managers in hospitals use patient's length of stay to measure hospital resource consumption and monitor the hospital performance [16]. The prediction of length of stay could help hospitals to estimate the forthcoming healthcare resource so as to increase the service level [16, 32]. The length of stay varies because of many factors including the patient's conditions, the types of diseases, the level of treatment, and the management of the hospital. The participators in healthcare activities all tend to shorten the length of stay nowadays in China. Clinical practitioners have the motivation to control the length of stay for certain diseases as a way to represent the level of treatment. For the managers of public hospitals, the limited bed resources push them to decrease the length of stay in order to receive more inpatients. With the popularity of modern healthcare knowledge, patients tend to stay shorter in the hospitals to minimize the risk of cross-infection. All the shortening tactics must be implemented on one base, which is to guarantee the treatment effect and the medical safety. Employing the length of stay in the configuration analysis could give an intuitive insight and guide the discussion in a more effective way.

### 3.2. Data Collections

Collaborating with a general public hospital in China, we obtain a dataset with 164121 medical records of inpatients. The data is generated from the EMR system in the year of 2015. The hospital stores the medical records in the electronic way ever since 1994, while the implementation of the EMR system was as late as in year 2013. This hospital adopted a structured multimedia EMR system, leading its way to adapt to the big data environment. EMR system in the hospital enables healthcare providers to create and retrieve patients' information in an effective and efficient manner. For the consideration of risk in the complex system, doctors and nurses somehow stick to using traditional paper ways to store inpatients information other than the paperless way [33]. So the integrity of the dataset in past years cannot be guaranteed. Understanding the pattern of learning and adapting of a new complex system by the doctors and nurses, we select the records of year 2015 for further analysis. After deleting records with missing values, we finally get 149633 available records.  Table 1 presents the characteristics of research dataset.

### 3.3. Results

The concept of configuration is similar to cluster or gestalts. Therefore, the method that forms configurations could be, but not limited to, cluster analysis or discriminate analysis. In this study, we first use cluster analysis to generate four configurations and then validate them through discriminate analysis. We utilize *K*-means to conduct the cluster analysis as this method could deal with a large amount of records in the big data environment [34] and standardize all data before clustering [35]. We also notice that the hospital cost and the length of stay are significantly different between surgical and nonsurgical inpatients [17]. Then we make a comparison between surgical and nonsurgical group to provide more insights of our findings.

**Figure 1:**
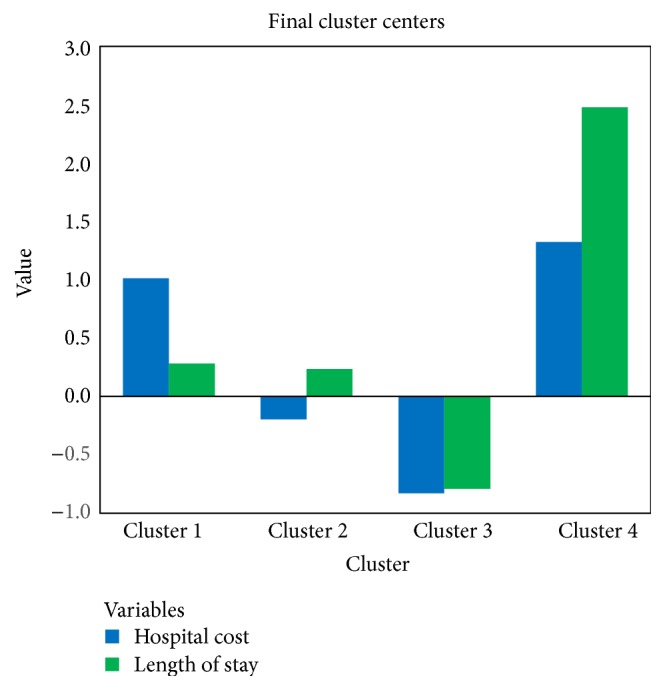
Cluster results.

We apply the standard algorithm of *K*-means clustering method. This method, acting as the nearest centroid classifier, could deal with massive data in a very short time. In the cluster analysis, we find that four groups could be generated and provide useful insights. All statistical values indicate the good validity of this result. After six iterations, the maximum absolute coordinate change for any center becomes zero.  Table 2 presents the number of cases in each cluster. Moreover, the *F* value in the ANOVA analysis is significant indicating that the values of total hospital cost and length of stay are significantly different between each cluster.  Figure 1 shows the value of key variables of each cluster center. We could notice that cluster 3 contains the largest number of cases. The hospital cost and the length of stay in cluster 3 are both low. Cluster 4 contains the lowest number of cases, while the hospital cost and the length of stay are both high. In cluster 1, the hospital cost is high while the length of stay is relatively low. In cluster 2, the hospital cost is low while the length of stay is relatively high.

In order to know the level of influence of each variable in determining which cluster the case belongs to, we conduct the discriminate analysis to find more insights. As three variables are needed in the discriminate analysis to create three functions for four groups, we add the age of inpatient as a personal factor. Age is known as an important factor in the personal level study [36]. Children and the aged people may face a higher risk of getting sick. Among the four characteristics mentioned before, age is the most common personal factor that could affect the treatment. That is why the DRGs grouping process selects the age of inpatient rather than other personal information as an influence factor. SPSS 20 is used for the discriminate analysis.  Table 3 presents the standardized canonical discriminate function coefficients. The results in Table 4 indicate that those three functions can significantly discriminate all the cases into four groups and the mean value of each group is also significantly different. Therefore, we find a way to predict which cluster the new case belongs to with the age of the patient, the length of stay, and the hospital cost.

**Figure 2:**
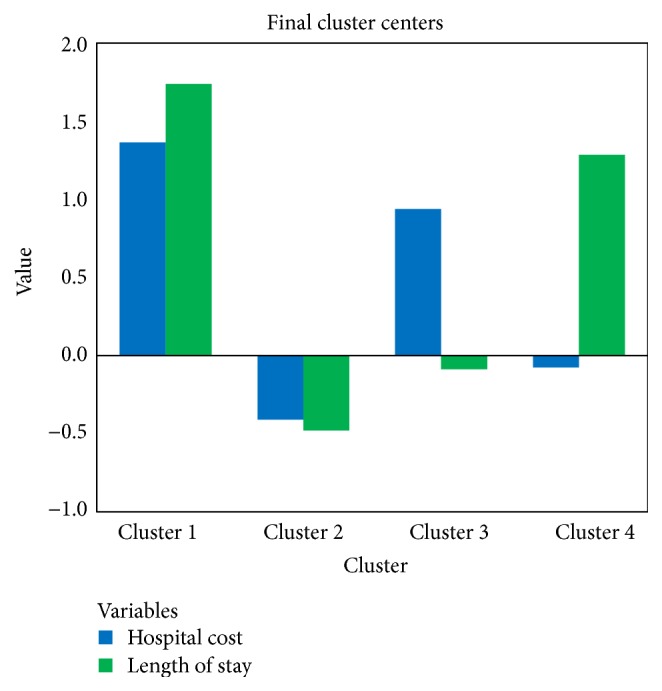
Cluster with surgical patients.

**Figure 3:**
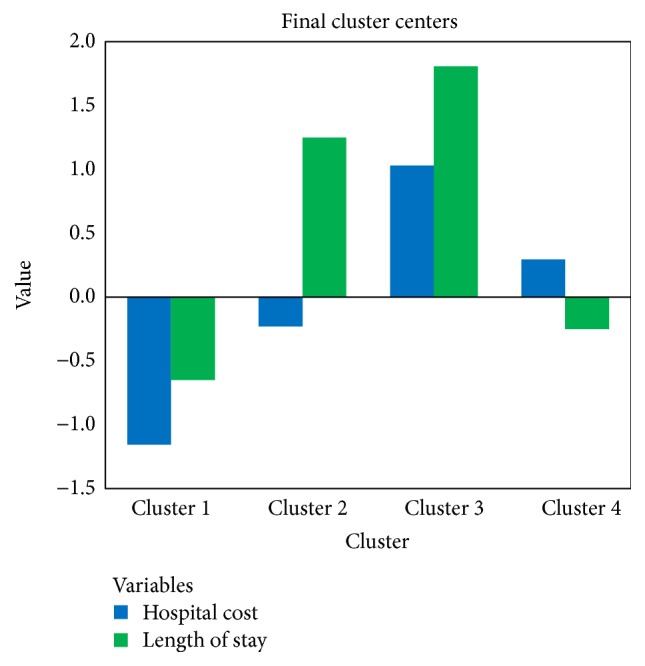
Cluster with nonsurgical patients.

We mark the cases 1 if its surgery cost is above 0. Two research groups are generated. In our dataset, 72086 patients underwent operations. Then, following the steps discussed above, we generate two sets of clusters for surgical and nonsurgical inpatients (see Figures 2 and 3). Tables 5 and 6 present the number of cases in each set of clusters. From the results, we can see that four distinct clusters are found within both surgical and nonsurgical patients. For each paired cluster (for example, cluster 1 in the surgical group and cluster 3 in the nonsurgical group), the hospital cost and length of stay are higher among surgical patients. Though differences exist in the level of hospital cost and length of stay, the four clusters show similar features. The paired cluster with both low hospital cost and low length of stay, cluster 2 in the surgical group and cluster 1 in the nonsurgical group, covers the largest population of inpatients. The paired cluster with low hospital cost and high length of stay, cluster 4 in the surgical group and cluster 2 in the nonsurgical group, contains the least population.

## 4. Discussion

**Figure 4:**
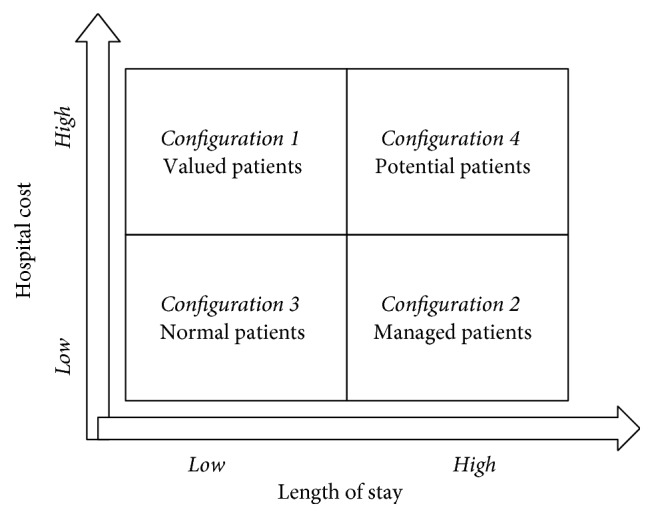
Inpatient configurations.

The findings of this study indicate that four clusters exist in inpatients' medical records. Based on value differences of each clusters, we identify four configurations and name them the valued patients, the managed patients, the normal patients, and the potential patients (Figure 4). Each inpatient configuration is named based on the common characteristics of inpatients from a managerial perspective. In this way, managers in the hospital could ignore the medical name and focus on the managerial features of inpatients. Therefore, strategies should be different when hospitals coordinate with inpatients in each configuration.

### 4.1. Configuration 1: Valued Patients

In this configuration, patients experience high cost and spend a relatively short time in the hospital. More than 2/3 of the patients in this configuration are treated with surgeries to get healed. The reason why this configuration is named valued patients is that, for hospital, great amount of values can be created in a relatively short time. When examining the data in this configuration with more details, we discover that almost 10% of the surgical patients are admitted by the same clinical department. Despite the characteristics of the diseases that this specialty treats, the administration of patients in this department should be studied to discover the secret of high efficiency. The waiting time at each process during the stay of every patient is curtailed to minimum to eradicate waste, which is the core principle of the management of Mayo Clinic. Many hospitals now have day-care units. The underlying philosophy is to lose some of the low-profit yet resource-consuming revenues such as nursing care and bed fees and to intensively concentrate on high-margin activities like surgeries. It could be deployed to the management of the valued patients to achieve the goal of generating high values and shortening the length of stay with limited bed and human resources. Administrators of the hospital should also explore the possibility of extending the experience to other specialties of similar features. With more cases going into this configuration, the whole operation efficiency, especially the economic benefits, of the hospital could be improved. However, it should be noticed that the basic purpose to set up a hospital is to bring healthy life condition to the patients. While endeavoring to gain values and shorten the length of stay, hospitals should never neglect the importance of treatment outcome. The rate of unscheduled return is an objective and sensitive index to evaluate the quality of treatment, which should be given close attention to in the management of valued patients.

### 4.2. Configuration 2: Managed Patients

Managed patients configuration means that the patients in this configuration should be paid more attentions in the care coordination activities. From the hospital's point of view, these patients possess the medical resources such as beds and nursing care without making full use of them while generating relatively low income. The number of surgical and nonsurgical patients in this configuration are comparatively equal. We would discuss the management of the two kinds of managed patients separately.

Most of the nonsurgical patients in this configuration are with chronic diseases such as arteriosclerotic heart disease, hypertension, or diabetes. They usually suffer from a serious acute attack and have to come to the hospital to recover. For the wellbeing of these patients, the diagnosis and treatment must be timely and rightful and the performance of the rescuing could be judged without waiting too long. Until the ending of the breathtaking rescue, the use of medical resources is at the high level. The low efficiency part of the treatment happens afterwards during the long recovering process when the doctors monitor the conditions of the patients to decide whether they are qualified to get discharged. This is a part where general public hospitals could cooperate with community medical institutes to efficiently utilize the bed and nursing resources there. When the state of the illness is relatively steady and controllable, inpatients of this configuration get treated and recover in these community medical institutes and transfer to general public hospitals when necessary. Telemedicine techniques should be employed to consolidate the cooperation [37]. Doctors with more sophisticated knowledge and experience in general public hospitals could do the ward round remotely and make suggestions to the following treatment methods. Doctors in community medical institutes could consult the opinions of doctors from the general public hospitals in hard decision-making or disagreement.

Surgical patients in the managed group suffer from nonfatal diseases in organs such as eyes and ears or in the respiratory or digestive systems. Normally the treatment of these diseases is standardized and the length of stay is controllable. The reasons that case goes to the managed configuration are from both the patient's side and the hospital's side. The conditions of the patient, including the age, the existence of the comorbidities, and complications, could all affect the time consumed to recover. The quality of healthcare provided by the hospital impacts the length of stay more profoundly. Any minor mistakes such as improper suture or untended tumble could severely delay the discharge of the patient. To decrease the number of cases in the managed configuration, which represents inefficient operation, hospitals can never be too precautious on preventing accidents from happening.

### 4.3. Configuration 3: Normal Patients

Normal patients experience a relatively low cost and a short period of stay in the hospital. This group contains the largest amount of cases, among which 2/3 are nonsurgical patients. Due to the large amount of patients, the sum of cost and time in this cluster is a number that cannot be ignored. Based on the Pareto principle, the proper management of the normal patients could largely protect the steady operation of the hospital. When studying the data with more details, we discover that nearly 13% of the cases are follow-up patients. Although follow-up patients often stay for no more than 3 days, the total consumption of bed and care with so large population is enormous. Other approaches to do the follow-up are recommended to explore. The necessity of admitting the patient to the hospital should be assessed. Inpatients with a negative result should be transferred to outpatients in the treatment schedule. For the other half that have to stay in the hospital, the utilization of family sickbeds could be an effective method to control the time and effort spent. With the aid of the remote medical technologies and the medical social networks, the knowledge flow from the hospital to the patients could be increased and medical safety of the family sickbeds could be realized and guaranteed. The rest of the normal patients are ill with clinically common diseases. The treatments are relatively standardized and can be managed in the clinical pathways. On one hand, the hospital should encourage more cases of each clinical pathway and optimize the treatment continually. On the other hand, medical quality and security should be given great attention when managing the normal patients, since complication and accidents could severely impact the cost and the length of stay and push the case to the managed configuration.

### 4.4. Configuration 4: Potential Patients

Potential patients spend a lot of time and money on the healthcare service. Patients in this cluster usually suffer from severe and complex diseases such as congenital heart disease or malignant tumors. Patients in the vegetative state relying on the medical instruments to survive also belong to this configuration. More than 70% of the patients in this group are treated with surgeries and all of them have the potential to cut the cost and time when healthcare technology develops. In order to offer a better service for patients in this cluster, the hospital should encourage more exploration on developing new techniques and treatments. The acts include, but not limited to, spending more efforts in the labs, cooperating with research institutes, and training special medical team for a specific disease. The scientific research in translational medicine should be greatly emphasized in treating these patients. While behaving the new techniques and treatments, healthcare providers must ensure the safety of the patients. Instances such as blinding, crippling, or even death have happened. Although the unfortunate results are somewhat inevitable, the potential risks and possible outcomes should be fully and thoroughly explained to the patients and admissions must be gained to perform the new techniques. Long period of stay and continually increasing cost could lead to potential risks of patients' complaining of the too high expense. To deal with the patients in this configuration efficiently and effectively, the hospital should make treatment orders and details of charges available to the patients. In this way, the satisfaction of the healthcare service could be increased.

## 5. Limitation and Future Research

Our findings on inpatient configurations offer some useful operations for the hospital from the managerial perspective. However, several limitations exist in this study. First of all, the results of our findings are limited to only one Chinese hospital. The pricing strategy of healthcare service, the consumption level, and public policy vary from different regions, which may affect the configurations and related strategies. The hospital ranked the 12th in 2016, which means the level of treatment and management is comparatively high. So the strategies applied to this hospital may not be realistic for other secondary-level hospitals. Secondly, this study just uses the medical records in the year of 2015. As the development of the Chinese society, factors that affect inpatient and hospital operations change rapidly. Therefore, configurations should be developed dynamically. Future research could also compare the differences among different years. Thirdly, the use of total hospital cost cannot indicate information about whether inpatients spend more on drugs and instruments or healthcare service. Strategies can greatly change with the influence of such information. Lastly, we did not analyze the effects of readmission and treatment outcomes on the length of stay in this study.

Our findings also show some directions for future research. First of all, future research can examine the dynamism of inpatient configurations with longitudinal data. Inpatient configurations could be different in different periods. For example, with huge increased family income or healthcare insurance, patients may require a higher level of services, which will significantly influence the length of stay and the hospital cost. Therefore, a set of periodically revised configurations will help hospitals gain more agile operations. Secondly, future research could add environmental factors such as the healthcare policy, the ecological environmental uncertainty, and the information intensity in the healthcare industry in the configuration analysis. These variables could enrich the analysis model and reveal more useful knowledge. From the results of the new configurations, hospitals may know which healthcare policy could improve the patient management and make related adjustments. Thirdly, future research could make comparisons on the inpatient configurations among different types of hospitals, especially between the general and specialized hospitals or between the top and secondary hospitals. The set of configurations could be viewed as a bunch of criteria, indicating the situation or quality of patient management of a hospital. Therefore, the results of those comparative analysis could guide hospitals to balance resources on their core patient configuration. Lastly, qualitative data could be added in future research to improve the explanations of our findings. In future research, face-to-face interviews with doctors, patients, and administrative staff of the hospital will reveal more information about each configuration. And it is also an efficient way to evaluate the results of the configuration analysis.

## 6. Conclusions

Based on a large amount of medical records from the EMR system, this study uses two key variables, the length of stay and the total hospital cost, to form a segmentation of inpatients with four configurations. With the information in the configuration analysis, we obtain new knowledge by examining the level of influence of each factor in predicting which configuration the inpatient belongs to. Moreover, our findings offer a way to study and manage the patients for hospital administration. Different strategies should be conducted consistently with different features in each configuration. Thereby, the healthcare service quality can be improved and the hospital operation can be optimized.

## Figures and Tables

**Table 1:** Sample characteristics.

	Frequency	Percent
*Sex*		
Male	78675	52.6
Female	70958	47.4

Total	149633	100.0

*Age*		
<21	18329	12.2
21–40	30816	20.6
41–60	63051	42.1
61–80	33665	22.5
>80	3772	2.5

Total	149633	100.0

*Marriage status*		
Single	28839	19.3
Married	110049	73.5
Widowed	1143	0.8
Divorce	537	0.4
Others	9065	6.1

Total	149633	100.0

*Total cost (CHN)*		
≤5000	24250	16.2
5000–10000	29629	19.8
10000–20000	41463	27.7
20000–50000	34648	23.2
>50000	19643	13.1

Total	149633	100.0

**Table 2:** Number of cases in each cluster.

Cluster	1	2	3	4	Valid
Cases	41816	30115	64669	13033	149633

**Table 3:** Standardized canonical discriminant function coefficients.

	Function		
	1	2	3
Length of stay	0.839	−0.653	0.016
Age	0.024	0.077	0.998
Hospital cost	0.327	1.004	−0.136

**Table 4:** Validity test in discriminant analysis.

Tests of equality of group means					
	Wilks's lambda	*F*	df1	df2	Sig.

Length of stay	0.333	100097.363	3	149629	0.000
Age	0.983	854.276	3	149629	0.000
Hospital cost	0.525	45122.144	3	149629	0.000

Wilks's lambda					
Test of function(s)	Wilks's lambda	Chi-square	df		Sig.

1 through 3	0.277	192059.008	9		0.000
2 through 3	0.889	17687.810	4		0.000
3	0.995	813.769	1		0.000

**Table 5:** Number of cases in each of the clusters (surgical patients).

Cluster	1	2	3	4	Valid
Cases	19083	32116	19725	1162	72086

**Table 6:** Number of cases in each of the clusters (nonsurgical patients).

Cluster	1	2	3	4	Valid
Cases	40062	2327	7056	28102	77547

## References

[1] Liu S., Wang L. (2016). Influence of managerial control on performance in medical information system projects: the moderating role of organizational environment and team risks. *International Journal of Project Management*.

[2] Deng Z., Liu S., Hinz O. (2015). The health information seeking and usage behavior intention of Chinese consumers through mobile phones. *Information Technology & People*.

[3] Moores T. T. (2012). Towards an integrated model of IT acceptance in healthcare. *Decision Support Systems*.

[4] Peng G., Dey D., Lahiri A. (2014). Healthcare IT adoption: an analysis of knowledge transfer in socioeconomic networks. *Journal of Management Information Systems*.

[5] Adler K. G. (2004). Why it's time to purchase an electronic health record system. *Family Practice Management*.

[6] Lim S. L., Ong K. C. B., Chan Y. H., Loke W. C., Ferguson M., Daniels L. (2012). Malnutrition and its impact on cost of hospitalization, length of stay, readmission and 3-year mortality. *Clinical Nutrition*.

[7] Liu S. (2016). How the user liaison's understanding of development processes moderates the effects of user-related and project management risks on IT project performance. *Information & Management*.

[8] Hillestad R., Bigelow J., Bower A. (2005). Can electronic medical record systems transform health care? Potential health benefits, savings, and costs. *Health Affairs*.

[9] Tu H., Yu Y., Yang P. (2012). Building clinical data groups for electronic medical record in China. *Journal of Medical Systems*.

[10] Weiskopf N. G., Weng C. (2013). Methods and dimensions of electronic health record data quality assessment: enabling reuse for clinical research. *Journal of the American Medical Informatics Association*.

[11] Zhang H., Wei X., Zhu M. (2014). The influence of human factors on medical record first page filling, on HQMS data. *Chinese Medical Record English Edition*.

[12] Quentin W., Scheller-Kreinsen D., Blümel M., Geissler A., Busse R. (2013). Hospital payment based on diagnosis-related groups differs in Europe and holds lessons for the united states. *Health Affairs*.

[13] Terho H., Eggert A., Haas A., Ulaga W. (2015). How sales strategy translates into performance: the role of salesperson customer orientation and value-based selling. *Industrial Marketing Management*.

[14] Vogus T. J., McClelland L. E. (2016). When the customer is the patient: lessons from healthcare research on patient satisfaction and service quality ratings. *Human Resource Management Review*.

[15] Reynolds D., Davenport D. L., Korosec R. L., Roth J. S. (2013). Financial implications of ventral hernia repair: a hospital cost analysis. *Journal of Gastrointestinal Surgery*.

[16] Panchami V. U., Radhika N. (2014). A novel approach for predicting the length of hospital stay with DBSCAN and supervised classification algorithms. *Journal of Intelligent Computing Volume*.

[17] Taheri P. A., Butz D. A., Greenfield L. J. (2000). Length of stay has minimal impact on the cost of hospital admission. *Journal of the American College of Surgeons*.

[18] Kankanhalli A., Hahn J., Tan S., Gao G. (2016). Big data and analytics in healthcare: introduction to the special section. *Information Systems Frontiers*.

[19] Raghupathi W., Raghupathi V. (2014). Big data analytics in healthcare: promise and potential. *Health Information Science and Systems*.

[20] Thompson C. D. (2013). *Benefits and risks of Electronic Medical Record (EMR): an interpretive analysis of healthcare consumers' perceptions of an evolving health information systems technology [Ph.D. thesis]*.

[21] Lau F., Price M., Boyd J., Partridge C., Bell H., Raworth R. (2012). Impact of electronic medical record on physician practice in office settings: a systematic review. *BMC Medical Informatics and Decision Making*.

[22] Reid C. M. (2010). Electronic health records today. *EContent*.

[23] Buntin M. B., Burke M. F., Hoaglin M. C., Blumenthal D. (2011). The benefits of health information technology: a review of the recent literature shows predominantly positive results. *Health Affairs*.

[24] Ross M. K., Wei W., Ohnomachado L. (2014). Big data and the electronic health record. *Yearbook of Medical Informatics*.

[25] Hajikhani S., Tabibi S. J., Riahi L. (2016). The relationship between the customer relationship management and patients’ loyalty to hospitals. *Global Journal of Health Science*.

[26] Bostan S., Acuner T., Yilmaz G. (2007). Patient (customer) expectations in hospitals. *Health Policy*.

[27] Turner J., Hansen L., Hinami K. (2014). The impact of hospitalist discontinuity on hospital cost, readmissions, and patient satisfaction. *Journal of General Internal Medicine*.

[28] Bhattacharjee P., Ray P. K. (2014). Patient flow modelling and performance analysis of healthcare delivery processes in hospitals: a review and reflections. *Computers & Industrial Engineering*.

[29] Hall R. W. (2006). *Patient Flow: Reducing Delay in Healthcare Delivery*.

[30] Lyytinen K., Damsgaard J. (2011). Inter-organizational information systems adoption—a configuration analysis approach. *European Journal of Information Systems*.

[31] Hussey P. S., Wertheimer S., Mehrotra A. (2013). The association between health care quality and cost a systematic review. *Annals of Internal Medicine*.

[32] Garg L., McClean S., Meenan B. J., Millard P. (2011). Phase-type survival trees and mixed distribution survival trees for clustering patients' hospital length of stay. *Informatica*.

[33] Liu S. (2015). Effects of control on the performance of information systems projects: the moderating role of complexity risk. *Journal of Operations Management*.

[34] Cui X., Zhu P., Yang X., Li K., Ji C. (2014). Optimized big data K-means clustering using MapReduce. *The Journal of Supercomputing*.

[35] Liu S., Wang L. (2014). Understanding the impact of risks on performance in internal and outsourced information technology projects: the role of strategic importance. *International Journal of Project Management*.

[36] Deng Z., Mo X., Liu S. (2014). Comparison of the middle-aged and older users' adoption of mobile health services in China. *International Journal of Medical Informatics*.

[37] Liu S., Xia F., Zhang J., Pan W., Zhang Y. (2016). Exploring the trends, characteristic antecedents, and performance consequences of crowdsourcing project risks. *International Journal of Project Management*.

