# Corrigendum to “A Way to Understand Inpatients Based on the Electronic Medical Records in the Big Data Environment”

**DOI:** 10.1155/2019/4598179

**Published:** 2019-01-20

**Authors:** Hongyi Mao, Yang Sun

**Affiliations:** ^1^Economics and Management School, Jiujiang University, Jiujiang 332005, China; ^2^Union Hospital, Tongji Medical School, Huazhong University of Science and Technology, Wuhan 430022, China

In the article titled “A Way to Understand Inpatients Based on the Electronic Medical Records in the Big Data Environment” [1], a fund number was missing. The Acknowledgments section should be updated as follows:

This work was supported by the National Natural Science Foundation (NSFC) Programs of China [71661017, 71661018, and 91646113] and Jiangxi Provincial Soft Science Research Program of China [20161BBA10033, 20161BBA10031].
